# Intestinal cellular heterogeneity and disease development revealed by single-cell technology

**DOI:** 10.1186/s13619-022-00127-6

**Published:** 2022-09-01

**Authors:** Yalong Wang, Wanlu Song, Shicheng Yu, Yuan Liu, Ye-Guang Chen

**Affiliations:** 1 Guangzhou Institutes of Biomedicine and Health, Chinese Academy of Sciences, Guangzhou Science Park, Luogang District, Guangzhou, China; 2 Guangzhou Laboratory, Guangzhou, China; 3 The State Key Laboratory of Membrane Biology, Tsinghua-Peking Center for Life Sciences, School of Life Sciences, Tsinghua University, Beijing, China

**Keywords:** scRNA-seq, Gut, Heterogeneity, Enteritis, Colorectal cancer

## Abstract

The intestinal epithelium is responsible for food digestion and nutrient absorption and plays a critical role in hormone secretion, microorganism defense, and immune response. These functions depend on the integral single-layered intestinal epithelium, which shows diversified cell constitution and rapid self-renewal and presents powerful regeneration plasticity after injury. Derailment of homeostasis of the intestine epithelium leads to the development of diseases, most commonly including enteritis and colorectal cancer. Therefore, it is important to understand the cellular characterization of the intestinal epithelium at the molecular level and the mechanisms underlying its homeostatic maintenance. Single-cell technologies allow us to gain molecular insights at the single-cell level. In this review, we summarize the single-cell RNA sequencing applications to understand intestinal cell characteristics, spatiotemporal evolution, and intestinal disease development.

## Background

**Fig. 1:**
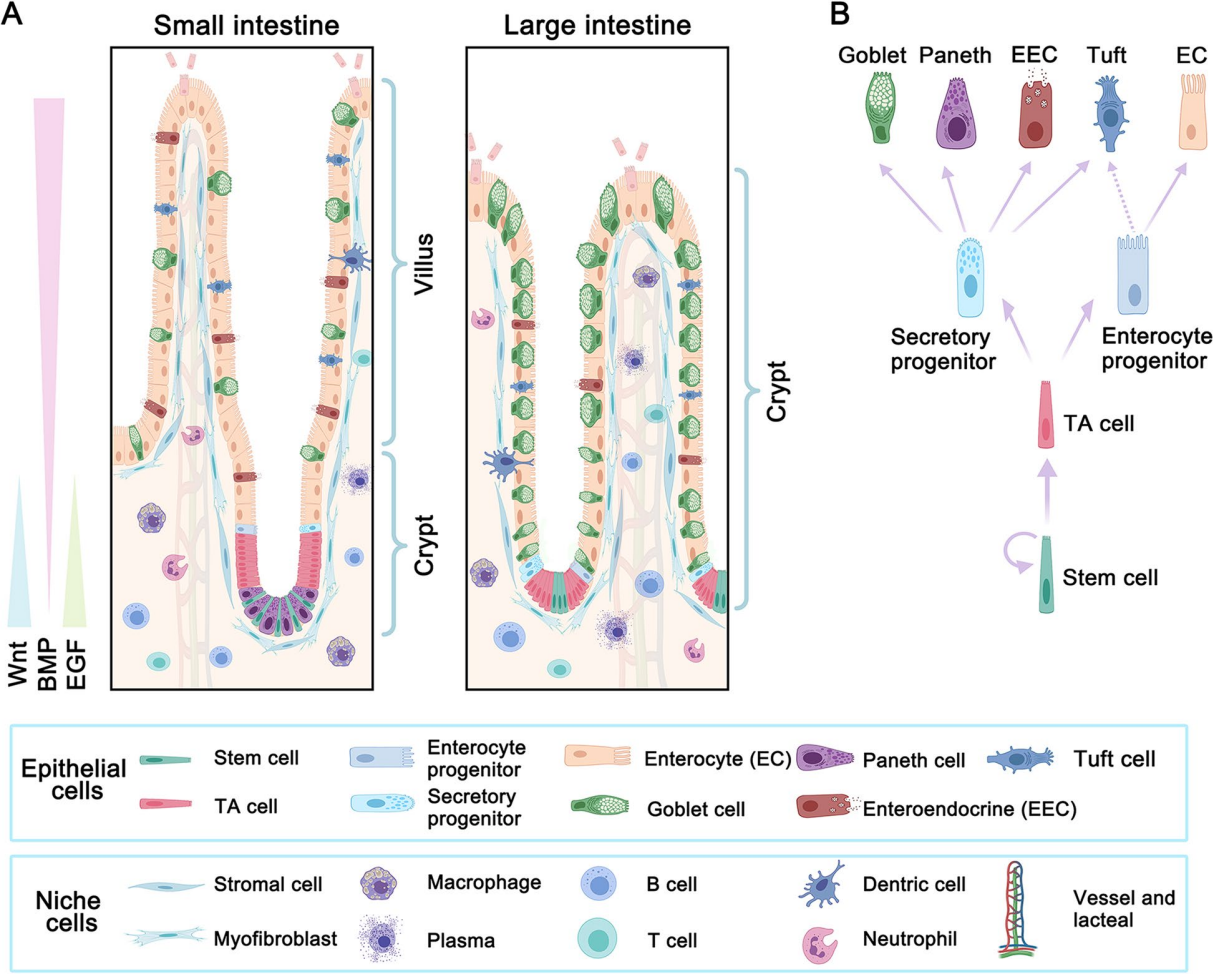
The structure and homeostatic maintenance of the intestinal epithelium. A, The small intestine is enriched with enterocytes and contains the crypt and villus structures, while the large intestine is enriched with goblet cells and only has the crypt structures. The cell constitution of both small and large intestinal epithelia is driven by Lgr5 + intestinal stem cells (ISCs) at the bottom of the crypt. The fate of ISCs is regulated by niche factors from the surrounding stromal cells or the epithelium cells themselves, such as Paneth cells. Wnt and EGF signaling promote proliferation, and their activities are high in the crypt and decrease gradually towards the villus. In contrast, BMP signaling promotes differentiation, inhibits proliferation, and induces cell death, and its activity increases gradually towards the villus tip. B, The proliferation and differentiation trajectory of intestinal epithelium. ISCs undergo self-renewal and meanwhile generate transit-amplifying (TA) cells. TA cells are fast proliferating and produce enterocyte and secretory progenitors, which further differentiate into enterocytes and Paneth, goblet, enteroendocrine and tuft cells, respectively. Tuft cells have also been suggested to derive from enterocyte progenitors

The intestine plays an important role in nutrient digestion and absorption, hormone secretion, protection and immune regulation (Peterson and Artis, 2014; Sanger and Lee, 2008; Zorn and Wells, 2009). Also, the intestine is the main organ for microbiota residing that has been showing continuously emerging functions to influence other organs in the body (Tilg et al., 2020). These functions depend on the intestinal epithelium that constitutes the second-largest epithelial surface area of more than 30m^2^ in the human body (Helander and Fandriks, 2014). The intestinal epithelial surface is expanded by millions of special structures, crypt-villus units in the small intestine or crypt units in the large intestine (Fig. 1A). The intestinal epithelium undergoes a fast turnover, which is driven by the intestinal stem cells (ISCs) located at the bottom of crypts (Fu et al., 2021). ISCs continuously self-renew and meantime generate transient-amplifying (TA) cells and progenitors, which then differentiate into mature functional cell types. Briefly, TA cells from ISCs give rise to secretory progenitors and enterocyte progenitors. Then the secretory progenitors further differentiate into goblet cells, enteroendocrine cells (EECs), Paneth cells, and perhaps tuft cells, while enterocyte progenitors differentiate into enterocytes (Fig. 1B). However, it has been reported that tuft cells may be from enterocytes but not secretory progenitors (Herring et al., 2018). During the differentiation progress, Paneth cells move down to the bottom of small intestinal crypts and are long-lived, whereas other mature lineages move up to keep the crypt or villus structures. These cell types cooperate with each other to form a multifunctional network. After the functional performance, these mature cells undergo cell death in a cycle of 4–5 days (Cheng and Leblond, 1974), and up to 10^11^ epithelial cells are lost every day in the human intestine (Leblond and Walker, 1956). This dynamic renewal system provides the intestine epithelium the ability to endure continuous chemical stimulation and various damages.

The dynamic balance of intestinal epithelium is maintained by the underneath niche cells and the microenvironment signaling (Zhu et al., 2021a). Most importantly, epidermal growth factor (EGF) and Wnt are abundant in the crypt to promote the proliferation of ISCs and TA cells, while bone morphogenetic protein (BMP) activity is higher at the villus, promotes cell differentiation and induces cell death (Fig. 1A) (Qi and Chen, 2015; Wang and Chen, 2018). These microenvironment signals are mainly from the niche cells, including stromal cells, fibroblast cells, immune-related cells, and others (Fig. 1A). For example, Forkhead box L1 (*Foxl1*) positive trophocytes can provide a source of Wnt-activation and BMP-inhibition signals for ISC self-renewal (Shoshkes-Carmel et al., 2018), and T helper cells can support ISC renewal through IL-10 (Biton et al., 2018). In short, the surrounding niche cells and signals are necessary for the epithelial function performance; the destruction of the microenvironment homeostasis may lead to intestinal disease development.

With the discovery of the specific ISC marker *Lgr5* (Barker et al., 2007), we have gained a great understanding of intestinal epithelium homeostasis in the last decade. A series of breakthroughs have been made in this field, such as intestinal organoid culture from single *Lgr5*^+^ cells (Clevers, 2016; Sato et al., 2009), stem cell contribution to intestinal tumorigenesis (Llado et al., 2015), and the repopulation of human colonic epithelium in vivo with potential therapeutic applications in the mouse model (Sugimoto et al., 2018). However, as in other scientific research fields, new findings beget new questions. For instance, the exact cell constitution and ratio of different cell types in small and large intestinal epithelia are unclear, the critical factors governing the fate trajectory of ISCs are not fully determined, and the cellular and molecular changes under intestinal pathological conditions are not well understood. In addition, cellular heterogeneity is an important theme for intestinal epithelium researches. For example, EECs can be divided into several subtypes (Gribble and Reimann, 2016), but the substantial crossover or the cellular heterogeneity is rarely revealed. Also, the mechanism of intestinal disease development is obscure, such as intestinal enteritis and colorectal cancer (CRC). These emerging questions require new advanced technologies to offer us a more comprehensive picture.

Although whole transcriptome analysis using bulk RNA-sequencing has yielded massive information on cell function and biological regulation, individual cells are the life identity and functional unit. Also, cellular heterogeneity is a general feature of biological tissues (Wen and Tang, 2016). To better understand the cellular composition and heterogeneity of primary tissues at the same time, single-cell PCR gene-expression analysis was developed about 10 years ago (Dalerba et al., 2011). Then, with the marked improvement of the single-cell RNA sequencing (scRNA-seq) technology during the past years (Grun et al., 2015; Magness et al., 2013), thousands of individual cells can be processed in a single experiment and more than 50,000 reads for each cell obtained (Chen et al., 2021b). Nowadays, scRNA-seq is widely used for routine transcriptional profiling at single-cell resolution (Zhao et al., 2021). Our cross-species analysis of the single-cell transcriptome of the ileum epithelium identified a new CA7 + cell type in pig, macaque, and human ileum and revealed the distinct expression pattern in enterocytes, EECs, and Paneth cells among different species (Li et al., 2022). In this review, we focus on an advanced understanding of the intestine biology gained from single-cell transcriptome analysis and provide an updated summary of the scRNA-seq database in the field.

## ScRNA-seq reveals cellular heterogeneity of the intestinal epithelium

Multiple functions of the intestinal epithelium are based on complex cell constitution with each cell type performing specific functions while coordinating with each other (Liu and Chen, 2020). Therefore, the identification of cell types is critical for understanding their detailed functions. Before the scRNA-seq technology application, identification of different cellular signatures at the transcriptome level was usually achieved by bulk RNA sequencing after fluorescence-activated cell sorter (FACS) sorting by cell-type specific markers (Table 1). However, bulk RNA-seq cannot reveal the cellular heterogeneity among these cells. ScRNA-seq makes it possible to detect the subtypes and their function difference at the single-cell level of the intestinal epithelium and the surrounding niche cells. More information about ISCs can be found in a recent review (Fu et al., 2021).

**Table 1:** Summary of the specific markers of intestinal epithelial cells

Cell types	Marker genes	
	**Mouse intestine**	**Human intestine**
**Enterocyte**	Alpi, Fabp1, Slc26a3 (Apoa1 in SI ^*^)	ALPI, FABP1, SLC26A3 (APOA1 in SI ^*^)
**Goblet cell**	Muc2, Spdef, Clca1, Zg16, Spink4, Fcgbp	MUC2, SPDEF, CLCA1, ZG16, SPINK4, FCGBP
**Enteroendocrine cell**	Neurog3, Neurod1, Chga, Chgb	NEUROG3, NEUROD1, CHGA, CHGB
**Paneth cell**	Lyz1, Defa5, Defa6	LYZ, DEFA5, DEFA6
**Tuft cell**	Dclk1, Pou2f3, Trpm5, Il25	POU2F3, TRPM5, IL25
**TA cell (Lgr5-negative)**	Mki67, Hmgb2, Top2a, Ube2c, Stmn1	MKI67, HMGB2, TOP2A, UBE2C, STMN1
**Stem cell**	Lgr5, Ascl2, Rgmb, Smoc2, (Olfm4 in SI ^*^)	LGR5, ASCL2, RGMB, SMOC2, (OLFM4 in SI ^*^)
**BEST4 cell**	Absent in mice	BEST4, SPIB, CA7, OTOP2
** + 4 stem cell**	Bmi1, Hopx, Tret, Lrig1, Mex3a	Need to be confirmed

^*^ *SI* small intestine

### Enterocytes

Enterocytes, a predominant cell type in the intestine, are responsible for food digestion and nutrient absorption, and these cells are derived from enterocyte progenitors (Pinto et al., 2003). Several transcript factors were found to determine the differentiation of TA cells to enterocyte progenitors (or immature enterocytes), such as *Hes1*, *Cdx2,* and *Hnf4* (Clevers and Batlle, 2013). Based on scRNA-seq, more transcription factors (*Sox4*, Foxm1, *Mxd3,* and *Batf2*) were identified to associate with enterocyte maturation in mouse small intestine (Haber et al., 2017). Similarly, *FABP1* and *KRT19* were found to participate in enterocyte maturation in the human small intestine (Fujii et al., 2018). In addition, a new subtype of enterocytes was found in the human colon, *BEST4*^+^ enterocytes, which comprise about 1% of the human ileal epithelium (Huang et al., 2019; Parikh et al., 2019; Smillie et al., 2019) and are enriched with the genes responsible for pH sensing and electrolyte transportation, including *GUCA2A*, *OTOP2,* and *CA7* (Brenna et al., 2015; Tu et al., 2018). These studies expand our knowledge about the functions of enterocytes, which are responsible for nutrient absorption and electrolyte balance.

### Goblet cells

Goblet cells synthesize and secrete mucins to prevent pathogen invasion and stabilize the bacterial biofilm (Zhang and Wu, 2020). Goblet cells are more enriched and contribute to two mucous layers in the large intestine: the outer layer is relatively loose and contains symbiotic bacteria, and the inner layer is tight and impervious to bacteria, while there is only one antibacterial gradient in the small intestine (Paone and Cani, 2020). The active mucin secretion in the large intestine is consistent with more goblet cells there compared with the small intestine (Wang et al., 2020b). The destruction of mucous layers and goblet cell functions may lead to enteritis development (Birchenough et al., 2016). ScRNA-seq trajectory analysis of mCherry-Muc2 cells showed that goblet cells segregate into two separate trajectories: one is enriched with canonical markers (*Clca1* and *Fcgbp*), while the other one is typically associated with enterocytes (*Dmbt1* and *Gsdmc4*) (Nystrom et al., 2021). This study also revealed a new subpopulation of goblet cells (intercrypt goblet cells, icGCs), which are especially located to the surface epithelium between crypts in the colon and contribute to a functional mucus barrier to protect the epithelium from microorganisms (Nystrom et al., 2021). Furthermore, scRNA-seq uncovered an immature goblet cell type, which is absent of *TBX10* expression, and was reduced during inflammation (Smillie et al., 2019). Another scRNA-seq study revealed that goblet cells could be divided into two main states: proliferation and differentiation. *HES6* was found in the early stage of goblet cell differentiation, which can be used to mark the goblet cells that are not yet morphologically identifiable as goblet cells (Zhang et al., 2019a). Similarly, mitotic goblet cells were identified by the co-expression of *MKI67*, *UBE2C*, *ZG16*, *TFF3,* and *CLCA1* in the human colon (Huang et al., 2019). Mature TFF1^+^ goblet cells are found only in the villus of the human ileum and in the top zone of crypts of the human colon and rectum, and they are highly enriched with the function of MHC antigen processing (Wang et al., 2020b). These results suggest that goblet cells show more heterogeneity along the differentiation trajectory.

### Paneth cells

Paneth cells, which are interspersed between *Lgr5*^+^ ISCs at the crypt base in the small intestine, secrete antimicrobial molecules modulating host-microbe interactions and provide the factors promoting *Lgr5*^+^ ISCs (Clevers and Bevins, 2013; Zhang and Liu, 2016). However, whether there are functionally equivalent cells in the large intestine is still under debate. With the application of single-cell technology, we have gained more insights into it. Single-cell PCR gene expression analysis identified a subset of *cKit*^+^ goblet cells in the mouse colon, which might have the equivalent function of Paneth cells in supporting *Lgr5*^+^ stem cells (Rothenberg et al., 2012). Furthermore, scRNA-seq of the human embryo’s digestive tract and adult intestinal segments unraveled the Paneth-like cells in human fetal and adult large intestine, respectively (Gao et al., 2018; Wang et al., 2020b). Interestingly, another scRNA-seq analysis revealed that *LYZ*, a Paneth cell marker, was upregulated in lower-crypt goblet cells and might mark the “deep crypt secretory cells” that are required to maintain the colonic stem cell niche and to protect stem cells from bacterial damage during colitis (Parikh et al., 2019). Moreover, scRNA-seq also uncovered the marker genes of Paneth cells in human ascending, transverse, and descending colons (Burclaff et al., 2022). Noteworthy, the cells designated as Paneth cells or Paneth-like cells in previous reports were designed as BEST4^+^ cells based on the expression of *LYZ*, *SPIB*, *BEST4,* and *CA7* (Burclaff et al., 2022; Wang et al., 2020b). Therefore, in addition to the canonical Paneth cells in the small intestine, the large intestine hosts cKit^+^/BEST4^+^/Paneth-like cells.

### Enteroendocrine cells

**Fig. 2:**
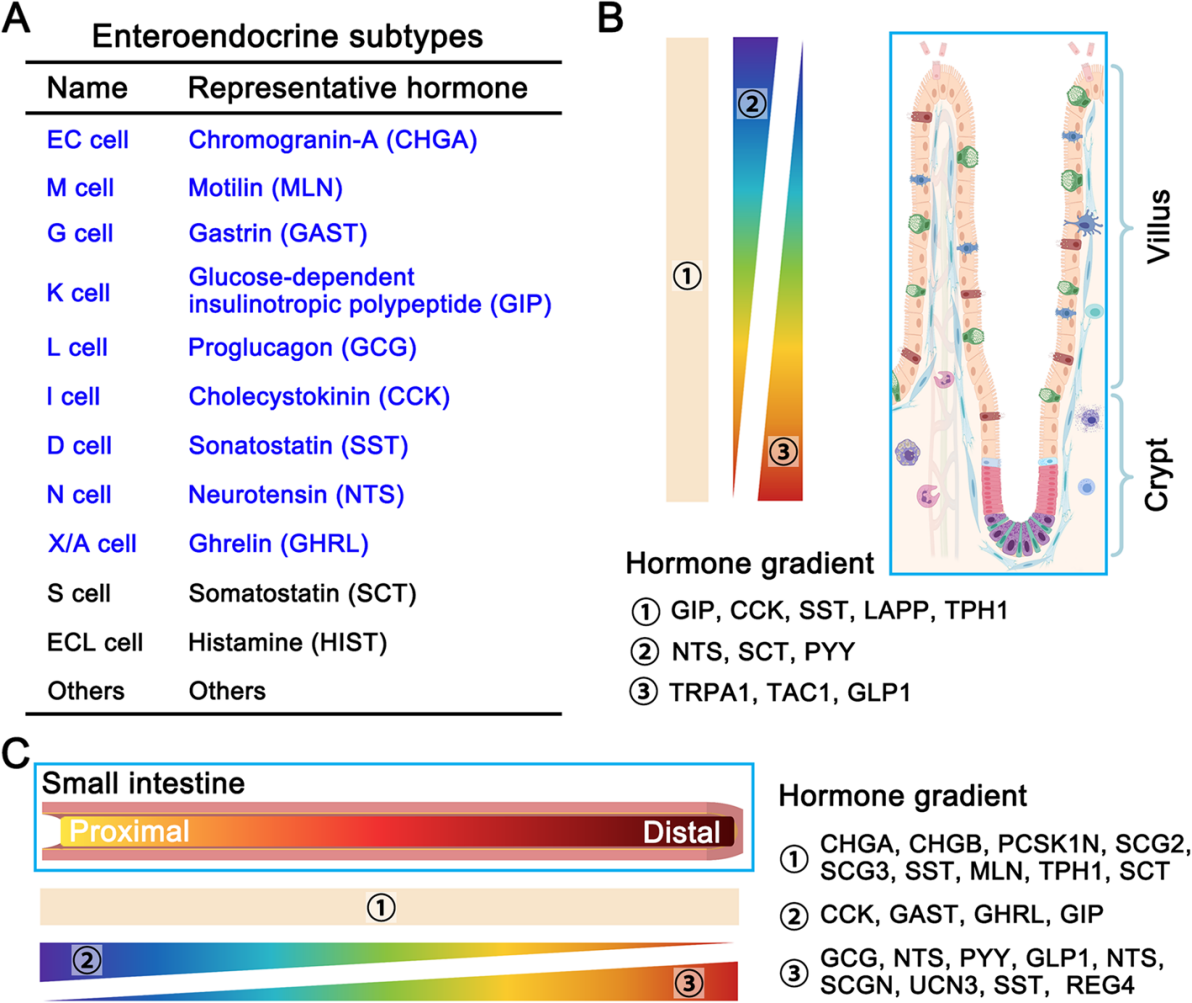
Enteroendocrine subtypes and their spatial distribution. A, Main subtypes of enteroendocrine cells and their secreted representative hormones (9 main subtypes in the human intestine are marked by blue). B, Secreted hormones show the spatial difference along the villus-crypt axis. TRPA1, TAC1, and GLP1 are enriched in the crypt, NTS, SCT, and PYY are enriched in the villus, while GIP, CCK, SST, LAPP, and TPH1 show no significant difference. C, Intestinal segmental enrichments of secreted hormones. CCK, GAST, GHRL, and GIP are enriched in the proximal segment, GCG, NTS, PYY, GLP1, NTS, SCGN, UCN3, SST, and REG4 are enriched in the distal segment, while CHGA, CHGB, PCSK1N, SCG2, SCG3, SST, MLN, TPH1, and SCT show no significant difference between the proximal and distal segments

EECs can sense nutrients and secrete about 20 different kinds of hormones (Furness et al., 2013). They have been divided into several subtypes based on hormone secretion (Gribble and Reimann, 2016). The scRNA-seq analysis of mouse intestine has uncovered hormone cross-expression among these subtypes. Haber and colleagues have re-defined the subtypes of mouse intestinal EECs into 12 sub-clusters and provided the hormones’ secretion atlas (Fig. 2A) (Haber et al., 2017). However, 9 sub-clusters EECs were assigned in the human small and large intestine in a single-cell transcriptomic atlas (Beumer et al., 2020): motilin (M cells), gastrin (G cells), gip (K cells), and cholestocystokin (I cells) were enriched in the duodenum, while glucagon (L cells) enriched in the colon. These results reveal different enteroendocrine subtypes between human and mouse intestines, including gene expression, such as *ASCL1* and *MNX1* in the human but not in mouse EECs (Beumer et al., 2020). A hormone gradient along the crypt-to-villus axis was revealed by scRNA-seq. For instance, tachykinin 1 (Tac1) and glucagon (Gcg, induced by Glp1) are highly expressed in the crypt, while secretin (Sct) and neurotensin (Nts) are high in the villus (Fig. 2B) (Beumer et al., 2018). Moreover, a hormone gradient is also observed along the small intestine from the proximal to distal segment: cholecystokinin (Cck) and gastrin (Gast) are enriched in the proximal segment, while proglucagon (Gcg) and peptide YY (PYY) enriched in the distal segment (Fig. 2C) (Beumer et al., 2020). Moreover, 10 major EEC subtypes were identified in adult *Drosophila* midgut, and these subtypes produce approximately 14 different classes of hormone peptides with each subtype on average secreting approximately 2–5 classes (Guo et al., 2019), demonstrating the complexity of the endocrine system in the intestine.

Along with the exploration of enteroendocrine subtypes, new marker genes are also identified by scRNA-seq. *Reg4* may be a novel marker for both *ChgA/ChgB*^high^ and *ChgA/ChgB*^low^ EECs (Grun et al., 2015) and *Neurog3*, *Neurod1,* and *Sox4* as new markers for enteroendocrine precursors (Gehart et al., 2019). ScRNA-seq also revealed that the EECs marked by *Bmi1* and *Prox1* could serve as a reservoir to replenish homeostatic and injury-inducible ISCs (Yan et al., 2017).

### Tuft cells

Tuft cells, marked by *Dclk1*, *Pou2f3*, *Trpm5*, *Il-25* (O'Leary et al., 2019), are rare solitary chemosensory cells in mucosal epithelia (Gerbe and Jay, 2016; Gerbe et al., 2012). These cells are involved in the initiation of immune type 2 responses (Gerbe and Jay, 2016) and produce a spectrum of biological effector molecules (Il-33, Il-25, prostaglandin D2, and the neurotransmitter acetylcholine) to mediate a cytokine-mediated cellular response (Gerbe and Jay, 2016; Schneider et al., 2019). Tuft cells closely interact with lymphoid cells (ILC2s) and recruit type 2 helper T cells (Th2 cells) to regulate immune activities in the intestine (Grencis, 2015; von Moltke et al., 2016). ScRNA-seq analysis has yielded new insights into tuft origin, differentiation progress, subtype, and functional subdivision.

Due to their secretion function, tuft cells were usually thought to originate from secretory progenitors that were regulated by *Atoh1* (Gerbe et al., 2012). However, using scRNA-seq, Herring and colleagues reported that tuft cells in the small intestine appeared distinct from the secretory lineage goblet and Paneth cells while showing a common trajectory with enterocytes (Herring et al., 2018). This study suggests tuft cells may not originate from secretory lineage. ScRNA-seq revealed that Dclk1-positive tuft cells express high levels of *Cox2* and *Hopx* in the mouse small intestine compared with the tuft cells in the colon (McKinley et al., 2017). ScRNA-seq studies also suggested that mature tuft cells may have two subtypes, one involved in the immune-related reaction and another related to neuronal development (Haber et al., 2017). These two subtypes present different enrichments in Il-33, the pan immune marker CD45, and the Th2-related cytokines Il-4 and Il-13 (Haber et al., 2017). Interestingly, tuft cell number increases after Paneth cell ablation and may act as novel niche cells in Paneth cell-ablated crypts (van Es et al., 2019). Moreover, the single-cell transcriptomic analysis showed that tuft cells may play an important role in intestinal enteritis—the enrichment of PSMA6 (Proteasome 20S subunit alpha 6, associated with Crohn’s disease risk) in tuft cells may lead to impaired epithelial integrity and stress responses (Huang et al., 2019).

## ScRNA-seq unveils spatiotemporal regulation

Along the crypt-villus axis, cells display multifarious states, and the same cell type may have specialized functions (Beumer et al., 2022; Moor et al., 2018). The functional diversity also exists from the proximal to the distal small and large intestine (Wang et al., 2020b). In other words, each cell could be precisely regulated, and the same type of cells may show cellular heterogeneity along with the spatiotemporal points. This spatiotemporal regulation makes the epithelium flexible to adapt to specialized functions and various environments such as nutrient constitution (Wang et al., 2020b), microbiota (Boulange et al., 2016), pathogens (Peterson and Artis, 2014), hypoxia (Zheng et al., 2015), chemical concentration changes (Williamson and Clifford, 2017) and chymopoiesis (Bhat et al., 2020). Spatial difference along the crypt-villus axis has been explored with bulk RNA sequencing (George et al., 2008; Mariadason et al., 2005), but the effect of cell heterogeneity and precise differentiation state cannot be taken into consideration. The studies based on scRNA-seq have provided insightful information on the spatiotemporal regulation of the intestinal epithelium.

Enterocytes exhibit significant functional differences along the crypt-villus axis according to scRNA-seq combined with RNA-seq of laser capture micro-dissected tissue (LCM-RNA-seq) (Moor et al., 2018). Amino acid and carbohydrate transporters were enriched in the middle of the villus, while the proteins involved in lipoprotein and chylomicron biosynthesis are mainly expressed at the top of the villus. Reg family members and other peptides involved in the microbiota-host interactions are highly expressed at the bottom of the villus. The spatially functional difference of enterocytes, such as the increased lipid uptake at the top of the villus and the Reg expression at the bottom of the villus, is confirmed by the analysis of scRNA-seq and Bmpr1a knockout mouse model, and the zonated gene expression is controlled by BMP signaling along the crypt-villus axis (Beumer et al., 2022). ScRNA-seq analysis also unraveled that enterocytes at the bottom and middle of the villus show more plasticity than the ones at the tip (Ayyaz et al., 2019).

Different intestinal segments exhibit distinct activities in nutrient absorption. ScRNA-seq analysis of the intestinal epithelium of the human ileum, colon, and rectum reveals the differences of signature genes and nutrient transporters in enterocytes among these three segments (Wang et al., 2020b). Although the genes related to protein digestion and absorption, mineral and organic substance transports are evenly expressed in all three segments, the genes participating in lipid metabolism and drug metabolic processes are highly expressed in the ileum, while the genes related to small molecule transport were enriched in the large intestine. The scRNA-seq analysis of the gene expression profiles of the human embryo digestive tract between 6 and 25 weeks of gestation showed that the genes involved in protein digestion and absorption were enriched in the small intestine compared with the stomach and large intestine (Gao et al., 2018). A large-scale single-cell spatiotemporal atlas of the human embryo digestive tract ranging from 8 to 22 post-conceptual weeks further revealed that the spatial distribution of absorptive genes is established in development prior to crypt formation (Fawkner-Corbett et al., 2021). Another spatial scRNA-seq of epithelial cells from the human duodenum to descending colon also showed the segmental absorption difference—fatty acid, glucose, and cholesterol transporters were enriched in the small intestine, while sodium transporters were enriched in the colon (Burclaff et al., 2022).

Like enterocytes, EECs also display spatial distribution in mice and humans based on scRNA-seq. EECs show differential hormone secretion in the villus (Sct, PYY, Nts) and crypts (Tac1, Glp1, Trpa1), while other hormones (Cck, Sst, Lapp, Tph1, Gip) show no significant difference along the crypt-villus axis in the mouse small intestine (Fig. 2B) (Beumer et al., 2018). Also, some hormones show segmental enrichment. The hunger-related hormones Ghrl and Gcg are enriched in the mouse duodenum, while PYY, an appetite reducer upon feeding, is found mainly in the mouse ileum (Fig. 2C) (Haber et al., 2017). In addition, some of the hormones are highly expressed in the human ileum (SCT, NTS, and CCK), while some are enriched in the human colon and rectum (PAM, NMB, and INSL5) (Wang et al., 2020b). Gip-producing K cells are enriched in the proximal part of the mouse small intestine, while Glp1-producing L cells in the distal segment, Sst-producing D cells, and enterochromaffin cells are uniformly distributed (Beumer et al., 2018). Consistent with the mouse results, GAST-producing G cells and CCK-producing I cells are enriched in the proximal segment, whereas NTS-producing N cells and GHRL-producing X cells are in the human distal small intestine (Beumer et al., 2020).

Regional transcription factors may contribute to EEC diversity according to scRNA-seq of the Drosophila midgut. Class-specific *Mirr* and *Ptx1* define Tk^+^ and AstC^+^ EEC types, respectively, while regional transcription factors contribute to segmental EEC identities. For instance, *Drm* defines the ITP^+^ EEC type in the posterior midgut, and *Esg* defines the NPLP2^+^ EEC type in the middle midgut (Guo et al., 2019). A time-resolution EEC subtype differentiation tree is given to depict common and lineage-specific transcriptional regulators during fate trajectory (Williamson and Clifford, 2017). Using a bi-fluorescent reporter (Neurog3-Chrono) to precisely position single-cell transcriptome along a time axis in mouse organoids, scRNA-seq identified temporal transcriptional regulators during enteroendocrine differentiation: *Neurog3*, *Dll1*, *C1qbp* function in the early stage, *Pax4*, *Insm1*, *Arx* in the intermediate stage, and *Isl1*, *Pax6*, *Elf4* in the late stage (Williamson and Clifford, 2017).

Like other organs, the intestine also undergoes structural and functional changes during organism aging (Pentinmikko and Katajisto, 2020), for instance, disruption of the intestinal barrier (Parrish, 2017), increased risk of inflammatory conditions (Shemtov et al., 2022), the loss of the regenerative capacity of ISCs and epithelial renewal (Funk et al., 2020; Mihaylova et al., 2018; Nalapareddy et al., 2017). Like other organs, the intestine also undergoes structural and functional changes during organism aging (Pentinmikko and Katajisto, 2020), for instance, disruption of the intestinal barrier (Parrish, 2017), increased risk of inflammatory conditions (Shemtov et al., 2022), the loss of stem cell plasticity and epithelial renewal (Funk et al., 2020). ScRNA-seq analysis of cultured mouse organoids further confirmed the reduced regenerative capacity in senescent ISCs and identified three TFs (*Egr1, Irf1, Fosb*) that were downregulated in aged ISCs and might account for 80% of the age-specific ISC transcriptome changes (Nefzger et al., 2022). The reduced canonical Wnt signaling activity in ISCs, Paneth cells, and mesenchyme may cause impaired ISC function upon aging (Nalapareddy et al., 2017). Consistently, the number of crypts and TA cells were decreased in the aging mouse intestine, which may be driven by mTORC1 via a p38 MAPK-p53 pathway (He et al., 2020). ScRNA-seq analysis of aging mouse colon also revealed that aging might cause a shift from absorptive to secretory epithelial cells, thus contributing to age-associated intestinal disturbances, such as malabsorption (Sirvinskas et al., 2022).

## ScRNA-seq reveals the mechanisms underlying enteritis

The gastrointestinal mucosa is the largest immunological organ in the body and represents a challenging environment where a meticulous balance must be maintained between tolerance and immune response from the huge microbiota burden (Abraham and Cho, 2009). Breakdown of the symbiotic relationship between the intestinal commensal microflora and the mucosal immune system leads to enteritis, such as inflammatory bowel disease (IBD) and other intestinal inflammation (Rathinam and Chan, 2018). Crohn’s disease (CD) and ulcerative colitis (UC) as two major types of IBD, both of which show defects of the epithelial barrier, accompanied with goblet cell decrease in UC and increase in CD (McCauley and Guasch, 2015). Various immune cells have been implicated in the pathogenesis of IBD, including macrophages (Chikina et al., 2020; Steinbach and Plevy, 2014), T cells (Lutter et al., 2018), innate lymphoid cells (Vivier et al., 2018), dendritic cells (Steinbach and Plevy, 2014), and plasma cells (Buckner et al., 2014). IBD is also associated with other events, such as deregulation of BMP and Wnt signaling (Kinchen et al., 2018), tumor necrosis factor (TNF) enrichment (Gaujoux et al., 2019), extracellular matrix remodeling (Zhang et al., 2020b), and genome instability (Wang et al., 2020a). Although the mechanisms underlying enteritis pathogenesis are partly uncovered, more comprehensive analyses are needed. In this regard, scRNA-seq provides a new insight into defining the disease-associated cell states and their possible interactions.

### The altered immune response in enteritis development

**Fig. 3:**
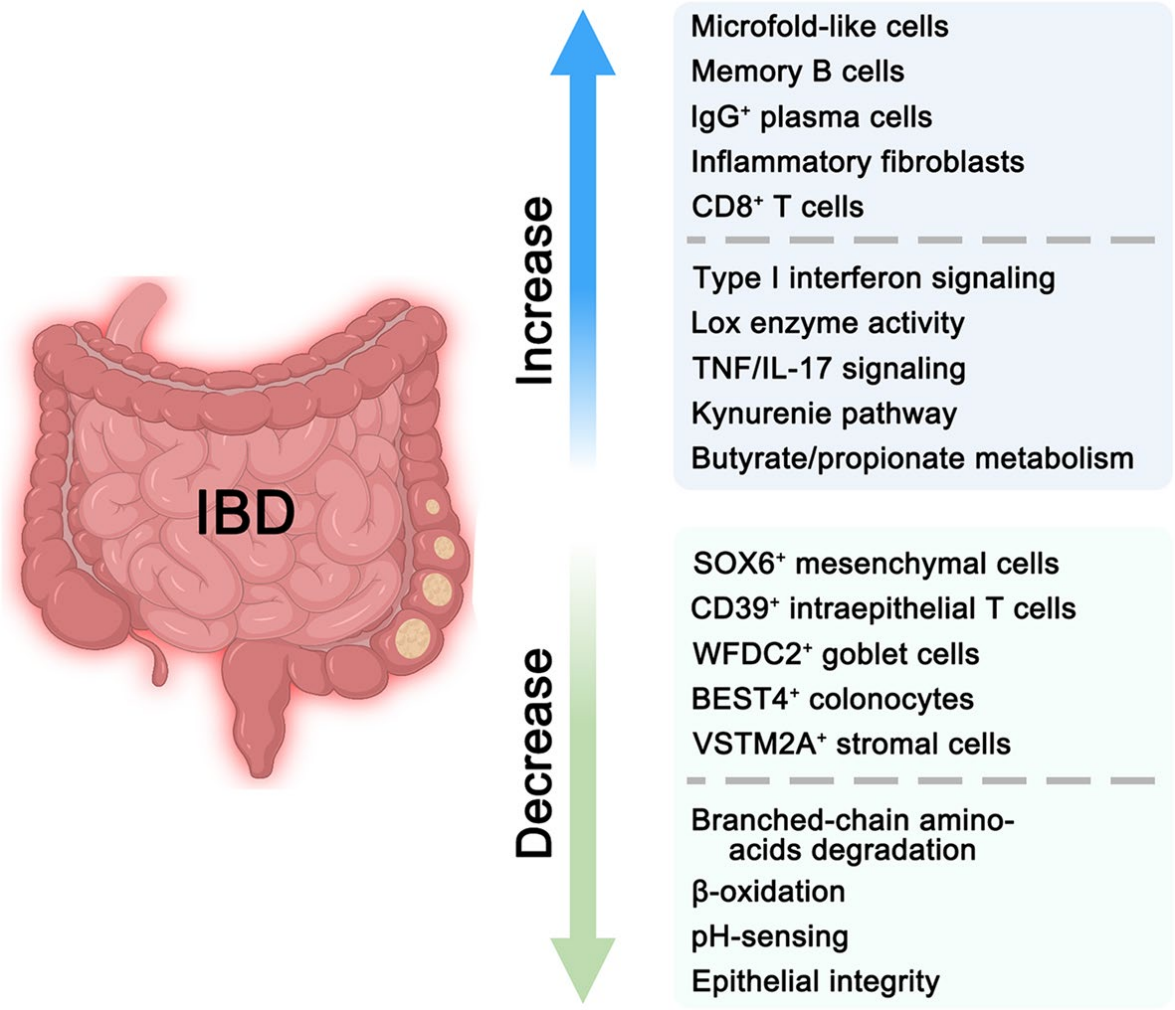
The alteration of cell types, signaling, and metabolism pathways in IBD. Some cell types are increased in IBD, such as microfold-like cells, memory B cells, IgG^+^ plasma cells, inflammatory fibroblasts, and CD8^ +^ T cells, while SOX6^ +^ mesenchymal cells, CD39^ +^ intraepithelial T cells, WFDC2^ +^ goblet cells, BEST4^ +^ enterocytes, and VSTM2A^ +^ stromal cell are decreased in IBD. Similarly, type I interferon signaling, Lox enzyme activity, TNF/IL-17 signaling, et al. are increased in IBD, while β-oxidation, pH-sensing, epithelial integrity, et al. are decreased in IBD

The intestinal immune system undergoes dramatic alterations during IBD development and is perceived as a key for effective treatment (Gorreja et al., 2022). Several unique T cell subsets were found by scRNA-seq from two severe CD cases, including *NKp30*^+^ γδT cells expressing RORγt and IL-26 (Jaeger et al., 2021). This study also revealed the increase of activated *CD8*^+^ T cells and the decrease of Treg cells in the inflamed regions (Fig. 3). Although the accumulation of *CD8*^+^ T cells was confirmed by another scRNA-seq analysis of immune cell populations in checkpoint inhibitor-induced colitis (CPI), the percentage of *FOXP3*^+^ Treg cells was found to be significantly elevated in CPI patients (Luoma et al., 2020), and the increase of special *FOXP3/BATF*^+^ Treg cells and *IL1B/LYZ*^+^ myeloid cells were confirmed by another study in inflamed UC patients (Devlin et al., 2021). Along with *CD8*^+^ T cells, *CD4*^+^ T cells were increased in CD patients, accompanied by an increased expression of the chemokines and cytokines, such as CXCL2, CXCL10, CXCL13, CCL11, and IL-6 (Elmentaite et al., 2020). Furthermore, the disease-specific patterns and metabolic changes were observed in *CD4*^+^ effector T cells according to the scRNA-seq analysis of 6 CD and 6 UC donors, including the increase of Toll-like receptor (TCR), Janus kinase (JAK), IL-17A, IL-22, TNF, et al. (Huang et al., 2021). Interestingly, *CD39*^+^ intraepithelial T cells are decreased in the pediatric IBD group, which may exacerbate colonic inflammation via platelet aggregation and 5-hydroxytryptamine (5-HT) release (Huang et al., 2019). These results confirmed the accumulation of activated *CD8*^+^ T cells during enteritis development, while the change of Treg cells was still unclear.

For other immune cell types, the expansion of IgG^+^ plasma cells, myeloid cells, and memory B cells were observed in the pediatric IBD group (Huang et al., 2019), and the increased IgG^+^ plasma cells and myeloid cells were also verified in CD patients (Elmentaite et al., 2020). Pathogenic expansion of IgG^+^ plasma cells and naïve B cells was also confirmed by scRNA-seq of human UC samples, which may be regulated by intestinal CXCL13-expressing follicular helper (TFH)-like T peripheral helper cells (Uzzan et al., 2022). Additionally, a shift from plasma cells to follicular cells and a decrease in the frequencies of IgA^+^ relative to IgG^+^ plasma cells were observed in the scRNA-seq survey of the 366,650 stromal cells from 18 UC patients and 12 healthy individuals (Smillie et al., 2019). Moreover, enrichment of mononuclear phagocytes was also observed in both infliximab- and vedolizumab-treated non-responder samples from IBD patients by scRNA-seq analysis, which was validated in the dextran sulphate sodium (DSS)-induced colitis mouse model (Liu et al., 2019). Therefore, most immune cells are increased in enteritis, especially for IgG^+^ plasma cells and myeloid cells, but other cell types, such as naïve B cells and mononuclear phagocytes, need further investigation. These studies illustrate the detailed change in the intestinal immune system during enteritis development.

### The association between environmental signaling and intestinal enteritis

Environment signaling also plays an important role in the pathogenesis of inflammation. Kinchen and colleagues surveyed the colonic mesenchymal atlas of health and IBD samples by scRNA-seq. They found that *SOX6*^+^ mesenchymal cells, which express TGF-β superfamily ligands BMP2 and BMP5, the non-canonical Wnt ligands WNT5A and WNT5B, and the Wnt antagonist FRZB, were decreased in inflamed UC colonic tissues (Kinchen et al., 2018). Similarly, PDGFRA, BMPs, WNT5A, SOX6, and matrix genes (*ADAMDEC1* and *GSN*) in the crypt top fibroblasts were decreased in human IBD tissues and mouse DSS model (Elmentaite et al., 2020; Kinchen et al., 2018). Another study showed that MAP3K2-regulated intestinal stromal cells are the source of R-spondin 1 following intestinal injury, which can protect the intestine from DSS-induced colitis in mice (Wu et al., 2021). These results illustrate the importance of the microenvironment remodeling in intestinal enteritis.

A common therapeutic target for IBD is TNF, which is usually upregulated during enteritis development (Kinchen et al., 2018). Integration of scRNA-seq and proteomics revealed that activation of integrin signaling is associated with anti-TNF therapy resistance in IBD (Brubaker et al., 2020). Enrichments of IgG plasma cells, inflammatory mononuclear phagocytes, and activated T and stromal cells are also associated with resistance to anti-TNF therapy according to the scRNA-seq analysis of human ileal CD samples (Martin et al., 2019). ScRNA-seq of NOD2-driven CD in zebrafish suggests that gp130 blockade could be used to complement anti-TNF therapy (Nayar et al., 2021). Therefore, upregulated TNF signaling, reduced activities of BMPs, and non-canonical Wnts may be associated with intestinal enteritis, which may guide therapy.

### Epithelial barrier and metabolism changes during enteritis development

The intestinal epithelium establishes the fundamental barrier for microbiota defense, intestinal luminal stress and mucosal immunity, and dysfunction of the epithelial barrier contributes to enteritis development. ScRNA-seq analysis of human colonic epithelia in health and clinically inflamed and noninflamed UC mucosa identified a novel pH-sensing absorptive colonocyte population marked by *BEST4/OTOP2* (Parikh et al., 2019), and its reduction was associated with IBD development (Fig. 3). A disease-associated cluster of goblet cells was also observed to highly express *WFDC2*, *KLF2*, *LAMC2*, *LAMB3*, *PLEC,* and *F3*, and these genes may play an important role in the integrity maintenance of the epithelial barrier (Parikh et al., 2019). In addition, other genes, which may contribute to pediatric IBD pathogenesis by causing impaired epithelial integrity and stress responses, were reported to be enriched in IBD epithelial cells, including *ERN1*, *PSMA6*, *DVL1*, *CASP7,* and *PIEZO1* (Huang et al., 2019).

Niche cells also contribute to the integrity maintenance of the intestinal epithelium. A stromal subset marked by *VSTM2A*, *SOX6,* and *AGT* was functionally related to the epithelial basement membrane at the physiological condition, and its deregulation contributes to epithelial barrier breakdown during colitis (Fig. 3) (Kinchen et al., 2018). Similarly, inflammation-driven fibroblasts could regulate mucosal matrix remodeling and healing in mouse DSS-induced colitis by producing IL-11 and the metalloprotease Adamdec1 (Jasso et al., 2022). The enrichment of fibroblasts and endothelial cells that participate in matrix remodeling and type I interferon signaling was also revealed by the single-cell transcriptomic analysis from the children samples with undifferentiated colitis, CD, and UC (Huang et al., 2019).

Interestingly, enteritis-associated inflammation also leads to metabolic changes in epithelial cells, such as induction of the kynurenine pathway and arginine biosynthesis enzymes, increased metabolism of butyrate and propionate, impaired degradation of branched-chain amino acids, and downregulation of β-oxidation (Fig. 3) (Smillie et al., 2019). The blockade of Lox enzyme activity in *Lox/Loxl1*^+^ mesenchymal cells could attenuate DSS colitis and reduce circulating markers of oxidative stress by reducing hydrogen peroxide (Kinchen et al., 2018). In addition, other changes may be pathogenic, such as 5-HT over-secretion (Huang et al., 2019), integrin signaling activation (Brubaker et al., 2020), and redox imbalances (Kinchen et al., 2018). Although the survey at the single-cell level surely advances our understanding of IBD development, the causal relationship between the dysfunction of the epithelial barrier, metabolism changes, and intestinal enteritis needs experimental verification.

### Mice as a model for human enteritis

DSS treatment or irradiation of the mouse intestine is often used as enteritis models (Zhu et al., 2021b). Assessment of the difference and similarity between mouse and human enteritis is important for the proper application of mouse models. ScRNA-seq analysis of colon and colitis showed that embryonic-specific genes reappear in response to intestinal damage, which is conserved both in human IBD and mouse DSS-induced colitis, including *MYO15B*, *S100A11,* and *CDV3* (Fazilaty et al., 2021). ScRNA-seq combined with spatial transcriptomic analyses also defined a subset of human IBD-risk genes that occur in the mouse DSS-treated colitis, such as the genes related to immune cell recruitment (e.g., Itgal, Icam1, Itga4), activation (e.g., Cd6, Plcg2, Ncf4, Il10ra), and antigen presentation (e.g., Tap1, Tap2, Psmb8) (Parigi et al., 2022).

However, different gene expression profiles were reported between human colitis and mouse model. For instance, *Serpina1c* (or *PI3* in humans) was only increased in mice after intestinal damage (Fazilaty et al., 2021). ScRNA-seq analysis also revealed that a new skin-like epithelial population called squamous neo-epithelium marked by the expression of *Sox2/Krt14/Krt7* could confer the resistance to colitis injury and rebuild the epithelial structure after colitis (Liu et al., 2022). This specialized population is only identified in the rectum of the DSS-treatment mouse model and need to be confirmed in human. These observations indicate that mouse colitis models should be cautiously used for human enteritis although some pathogenetic mechanisms are conserved.

## Single-cell technologies reveal novel insights into gut cancer development

Deciphering tumor heterogeneity is a key to get a comprehensive understanding of tumor origination and to find better and more precise treatments. Our current knowledge of intra-tumoral heterogeneity is largely gained from the analysis of bulk tumor specimens, including bulk DNA/RNA sequencing. However, most bulk tumor specimens consist of a mixture of nonmalignant cells and diverse subpopulations of cancer cells. To gain more insight into intra-tumoral heterogeneity, single cancer cell clones have been generated (Roerink et al., 2018). But these clones still cannot cover all the cancer cells with distinct behavior. In this regard, single-cell technology exhibits its advantage in dissecting the multiple dimensions of intra-tumoral heterogeneity and their evolutionary relations (Francis et al., 2014; Navin et al., 2011). Combining single-cell DNA sequencing (scDNA-seq) and scRNA-seq, more details can be obtained at the single-cell resolution, such as tumor cell heterogeneity (Darmanis et al., 2017), epithelial-to-mesenchymal transition (EMT) (Pastushenko et al., 2018), cancer metastasis (Puram et al., 2017), and tumor microenvironment (Neal et al., 2018).

### Transcriptomic heterogeneity of CRC

CRC development is influenced by numerous factors whose combinatory effects lead to various tumor subtypes and cellular heterogeneity (Dekker et al., 2019). ScRNA-seq has been used to profile various tumors and has revealed the cell constitution, tumor-initiating cells, and the microenvironment cells. For instance, it has been reported that stem-like cells accounted for 93% of tumor epithelial cells in CRC, but only 30% in normal mucosa epithelium, according to a new clustering method named reference component analysis (Li et al., 2017). This study also grouped colorectal tumors into subtypes with proliferating and differentiated states, which were previously assigned to a single cell type by bulk transcriptomics (Li et al., 2017; Wu et al., 2017). The distinct cell subpopulations resembling differentiation states of normal intestinal epithelial cells (ISCs-like, TA-like, and differentiated cells) were also revealed by other scRNA-seq analyses in CRC patients (Wu et al., 2017; Zowada et al., 2021). These ISCs-like and TA-like cells showed high levels of oxidative phosphorylation and mitochondrial membrane potential, which were linked to tumor-initiating activity (Zowada et al., 2021).

ScRNA-seq has also been employed to investigate the epithelial-mesenchymal interaction in CRC pathogenesis. Analysis of cancer stem cells in CRC in *Apc*^Min/+^ mouse adenoma at various time points during chemoradiotherapy suggests that tumor-initiating cells can shape the tumor environment into a landscape, which renders resistance to immunosuppression by forming an immune barrier against CD8^+^ T cells and promotes the proliferation of therapy-resistant cancer stem cells via Cox-2/PGE2 signaling (He et al., 2021).. Moreover, cancer stem cell activity is also regulated by the niche BMP and WNT signaling secreted by the *PDGFRA*^low^/*CD81*^+^ stromal cells (Yum et al., 2021). The oncogene reporter mouse model combined with scRNA-seq analysis suggests that oncogene-expressing crypts can replace neighboring wild-type crypts over time, thereby leading to accelerated clonal drift and CRC development (Yum et al., 2021). In summary, these results implicate that the transformed epithelial cells can accelerate the tumorigenesis process themselves and cultivate the environment to facilitate tumor development.

### The role of the immune system in CRC therapeutic outcomes

Apart from promoting tumor transformation of the intestinal epithelial cells, the immune cells also play an important role in CRC therapeutic outcomes and influence tumor metastases. ScRNA-seq transcriptomic profiling of T cells from peripheral blood, adjacent normal and tumor tissues of CRC patients showed that the *CXCL13*^+^/*BHLHE40*^+^ subset of *IFNG*^+^ Th1-like T cells was preferentially enriched in microsatellite instability tumors (Zhang et al., 2019b). Treatment of a CD40-activating antibody could expand Th1-like and *CD8*^+^ memory T cells in colon cancer (Zhang et al., 2020a). Furthermore, the scRNA-seq analysis of 37,931 T cells from 16 CRC patients suggests the phenotypically and functionally distinguishable *CD4*^+^ and *CD8*^+^ effector T cell types are associated with clinical outcomes (Masuda et al., 2022). The *GZMK*^+^/*KLRG1*^+^ cytotoxic *CD8*^+^ T cells with a less dysfunctional phenotype were enriched in CRC patients with good outcomes. For *CD4*^+^ T-cell infiltrates, Helios^+^ Treg cells were associated with good outcomes, while *Helios*^*−*^*/CD38*^+^ peripherally-induced Treg cells were strongly associated with bad outcomes. These immune cells may be good targets for advanced treatments and the prognosis of CRC.

It has been reported that specific macrophage and conventional dendritic cell subsets may be key factors in the development of colorectal adenoma and liver metastases, according to the scRNA-seq of 18 treatment-naive CRC patients (Zhang et al., 2020a). ScRNA-seq and spatial transcriptomic analysis further revealed that immune-suppressive cells, especially *MRC1*^+^/*CCL18*^+^ M2-like macrophages, were enriched in live metastases of CRC (Wu et al., 2022). Single-cell transcriptomic profiling of lung metastases from CRC patients uncovered that a special subtype of B cells (*ERBIN*^+^) was involved in cancer metastases, and targeting Erbin as well as the combinatory block of B cells and PD1 could suppress lung metastasis of CRC in mice (Shen et al., 2021). Therefore, special T cells may be associated with CRC development, and macrophages may play an important role in CRC metastases. These scRNA-seq results present a more comprehensive understanding of the immune roles in tumor development and metastasis and provide new insights to improve immune therapies.

### Single-cell DNA-seq confirms tumor heterogeneity

In addition to scRNA-seq, scDNA-seq has also exhibited its power in the understanding of cellular heterogeneity of CRC (Jiang et al., 2021; Navin et al., 2011). An early study of 63 single tumor cells identified the *SLC12A5* gene with a high frequency of mutation at the single-cell level but exhibited low prevalence at the bulk population level (Yu et al., 2014). By far, single-cell whole-exome sequencing of CRC has discovered many important gene mutations. One unique sub-clonal mutation of *CSMD1* was detected exclusively in the adenomatous polyps by scDNA-seq, but not by bulk sequencing (Wu et al., 2017). A similar scDNA-seq analysis also revealed mutations and somatic copy number alterations hidden in bulk sequencing (Liu et al., 2017). ScDNA-seq also revealed that *EpCAM*/*CD44* colonic cancer stem cells are critical for cancer development, metastasis, and drug resistance (Du et al., 2008; Went et al., 2004). Single-cell whole-genome sequencing of *EpCAM*^high^/*CD44*^+^ cancer stem cells and *EpCAM*^high^/*CD44*^−^ differentiated cancer cells in colon cancer indicated that those cells from the same patient had a similar somatic copy number variation pattern. However, the somatic copy number variation was found to be different in certain patients and might confer different growth advantages at later stages (Liu et al., 2018). Moreover, scDNA-seq of the primary tumors and liver metastases of CRC unraveled the extensive intra-tumoral heterogeneity at the primary and metastatic tumor sites, as well as the late dissemination of metastases from early progenitor clones (Leung et al., 2017).

Multiple techniques were employed to simultaneously assess somatic copy number variation, DNA methylation, and transcriptome of 1,900 single cells from 10 CRC patients at the single-cell level (Bian et al., 2018). The results uncovered that the genome-wide DNA demethylation patterns of cancer cells were consistent in all 10 patients, while the DNA demethylation degrees were correlated with the density of the heterochromatin-associated H3K9me3 of normal tissues (Bian et al., 2018). Also, combined analyses of scDNA-seq and scRNA-seq on tumor niche cells from 21 colorectal patients showed that somatic copy number alterations were prevalent in immune cells, fibroblasts, and endothelial cells in tumors compared with adjacent tissues (Zhou et al., 2020). Furthermore, five genes (*BGN*, *RCN3*, *TAGLN*, *MYL9,* and *TPM2*) were identified as fibroblast-specific biomarkers for the poor prognosis of CRC. ScDNA-seq and scRNA-seq were also applied to the organoids, which were derived from single normal intestinal cells, subjected to RNAi-mediated downregulation of the tumor suppressor gene APC, and then transplanted to mice to investigate how intra-tumoral heterogeneity is generated. The results showed that the emergence of new transcriptional subpopulations was the key for the adaptation of cancer cells to drastic microenvironmental changes (Ono et al., 2021). All these studies clearly demonstrate that the single-cell studies provide novel insights into cancer initiation, metastases, heterogeneity, and relation to the microenvironment, and would advance disease diagnosis and treatment.

## Conclusions and Perspectives

With the development of single-cell technology, we have gained a more comprehensive understanding on the intestinal cellular constitution, characterizations, and heterogeneity (Table 2). Single-cell sequencing has also yielded new insights into the pathogenesis of enteritis and intestinal cancer. Although a significant advance has been made, many questions remain. Single-cell transcriptomic analysis has unraveled new subtypes of intestinal tuft cells, enterocytes, and EECs, but the heterogeneity of other cell types and the landscape of the intestinal immune system need further investigation. Epithelial progenitors have been divided into absorptive and secretory subtypes (Clevers and Batlle, 2013), but no clear subpopulations are reported. It may be due to a vague definition of progenitors and unclear differences between progenitors and TA cells. Attention has also been paid to the differences between *Lgr5*^high^ and *Lgr5*^low^ subpopulations (Baulies et al., 2020), but the heterogeneity among intestinal stem cells is still unclear. Even the dispute over whether there are quiescent stem cells or + 4 stem cells is not settled. Microfold cells are indicated to be essential for antigens transport to the lymphoid cells underneath (Mabbott et al., 2013), but the atlas of microfold cells is rarely known because of their low cell number in the intestinal epithelium. The spatial heterogeneity of enterocytes and EECs has been explored along the crypt-villus axis and in different intestinal segments (Beumer et al., 2018; Wang et al., 2020b). More comprehensive investigations on spatial cell heterogeneity, pathophysiological significance, and the underlying mechanisms are needed. Also, the spatiotemporal transcriptional atlas of EECs has been reported. Similar studies should be applied to other epithelial cell types and niche cells during intestine development and regeneration, which would help address the question concerning what regulates the formation of the special structures. For instance, Hedgehog signaling has been reported to be critical for villus formation (Rao-Bhatia et al., 2020).

**Table 2:** Summary of the scRNA-seq studies in the intestine

Organism	Sample type	Sample source	Cell type	Library preparation method	Cell number	Data resource	Reference
*Mus musculus*	Jejunum	Labeled stem cells	Short-term and long-term label-retaining stem cells	Fluidigm	558	Supplementary Table 2	(Li et al., 2016)
*Mus musculus*	Jejunum	Wildtype mice	Epithelial, mesenchymal and immune cells	MARS-seq	329	GSE134479	(Bahar Halpern et al., 2020)
*Mus musculus*	Jejunum	Stem cells	Prox1-GFP^+^, Bmi1-GFP^+^, Lgr5-eGFP^+^, and Lgr5-eGFP^−^ cells	10X Chromium	3,521	GSE99457	(Yan et al., 2017)
*Mus musculus*	Ileum	Massive small bowel resection	Epithelial cells	10X Chromium	19,245	GSE130113	(Seiler et al., 2019)
*Mus musculus*	Small intestine	Wildtype mice	Crypt cells	C1	76	GSE146783	(Sato et al., 2020)
*Mus musculus*	Small intestine	Labeled epithelial cells	Bmi1, Hopx or Lgr5 labeled cells	Fluidigm	1,033	Supplementary Table 2	(Li et al., 2014)
*Mus musculus*	Small intestine	Labeled epithelial cells	Intestinal preproglucagon-expressing cells	Smart-seq2	288	Not described	(Glass et al., 2017)
*Mus musculus*	Small intestine	Wildtype mice	Epithelial cells	10X Chromium	7,216	GSE92332	(Haber et al., 2017)
*Mus musculus*	Small intestine	Stem cells	Lgr5^high^ cells	Smart-seq	245	GSE90856	(Barriga et al., 2017)
*Mus musculus*	Small intestine	Stem cell depleted and normal epithelium	Epithelial cells	10X Chromium	192	Not described	(Tetteh et al., 2016)
*Mus musculus*	Small intestine	Lgr5-eGFP^+^ intestinal stem cells	Stem cells	10X Chromium	13,247	GSE92865	(Yan et al., 2017)
*Mus musculus*	Small intestine	Wildtype and irradiated mice	Epithelial cells	10X Chromium	6,644	GSE123516	(Ayyaz et al., 2019)
*Mus musculus*	Small intestine	Small intestine mucositis	Epithelial cells	10X Chromium	12,653	GSE131630	(Zhao et al., 2020)
*Mus musculus*	Small intestine	Wildtype and Lats1/2 knockout mice	Pdgfrβ^+^ intestinal stromal cells and lymphatic endothelial cells	10X Chromium	7,906	GSE124488	(Hong et al., 2020)
*Mus musculus*	Small intestine	Normal diet and high-fat/high-sugar diet mice	Epithelial cells	10X Chromium	27,687	GSE147319	(Aliluev et al., 2021)
*Mus musculus*	Small intestine	Wildtype mice and β7-KO mice	Epithelial cells	10X Chromium	13,352 for WT; 10,763 for β7-KO	OEP000370	(Chen et al., 2021a)
*Mus musculus*	Small intestine	Wildtype; Fltp^ZV/+^; Foxa2^FVF/FVF^ mice	Epithelial cells	10X Chromium	60,000	GSE152325	(Bottcher et al., 2021)
*Mus musculus*	Small intestine	Wildtype crypts in Red2-KrasG12D and Red2-PIK3CAH1047R mice	Epithelial, mesenchymal and immune cells	10X Chromium	21,183	E-MTAB-8656	(Yum et al., 2021)
*Mus musculus*	Small intestine	Wildtype mice	Epithelial cells	MARS-seq	Not described	GSE178586	(Zinina et al., 2022)
*Mus musculus*	Small intestine and colon	Casp3/7^ΔIEC^ mice and Casp3/7^FL/FL^ littermates	Epithelial cells and immune cells	10X Chromium	7,584 Casp3/7^ fl/fl^ cells and 11,956 Casp3/7^ΔIEC^ cells	GSE183885	(Ghazavi et al., 2022)
*Mus musculus*	Distal small intestine and colon	Muc2-mCherry mice	Goblet cells	10X Chromium	6123 cells for the colon and 3552 cells for the small intestine	GSE144436	(Nystrom et al., 2021)
*Mus musculus*	Colon	Wildtype mice	Gli1^+^ stromal cells	10X Chromium	4,464	GSE113043	(Degirmenci et al., 2018)
*Mus musculus*	Colon	LGR5^+^ lineage tracing and lethal injury (irradiation) mice	Regenerating crypt cells and Ascl2-deficient colonic stem cells	10X Chromium	3,254	GSE130822	(Murata et al., 2020)
*Mus musculus*	Colon	Wildtype mice	Pdgfra^+^ endothelial cells and epithelial cells	10X Chromium	6,358	GSE130681	(McCarthy et al., 2020c)
*Mus musculus*	Colon	NeuroD1-Cre x Rosa26-EYFP labeled mice	EECs	10X Chromium	1,560	Not described	(Billing et al., 2019)
*Mus musculus*	Colon	Wildtype mice	Epithelium, endothelial and stromal cells	10X Chromium	7,395	GSE151257	(Brugger et al., 2020)
*Mus musculus*	Colon	Wildtype mice	T cells	10X Chromium	7,012	GSE160055	(Kiner et al., 2021)
*Mus musculus*	Colon	DSS-exposed 3 days	Epithelial and mesenchymal cells	10X Chromium	11,495	GSE156245	(Wu et al., 2021)
*Mus musculus*	Colon	MC38 xenografts upon SHP099 treatment	Cancer cells, stromal cells, and immune cells	10X Chromium	7,934(SHP099), 7,881(PBS)	GSE164908	(Gao et al., 2022)
*Mus musculus*	Colon	Wildtype and DSS treated mice	Stromal cells	10X Chromium	34,197	GSE172261	(Jasso et al., 2022)
*Mus musculus*	Distal colon	Wildtype and DSS treated mice	Epithelial, stromal and immune cells	10X Chromium	> 35,000	GSE168033	(Liu et al., 2022)
*Mus musculus*	Colorectal cancer	Primary tumor and lung metastases	Immune cells	10X Chromium	12,588 B220^+^ cells, 3,748 CD38^+^ cells, 1,588 CD79a^+^ cells	Not described	(Shen et al., 2021)
*Mus musculus*	Small intestine, colon	Wildtype mice	Endothelial cells	10X Chromium	> 32,000	E-MTAB-8077	(Kalucka et al., 2020)
*Mus musculus*	Intestine	pLys^DTR^ mice (DT-treated for 6 consecutive days)	Paneth cells	CEL-seq	288	Supplementary dataset S01	(van Es et al., 2019)
*Mus musculus*	Intestine	Wildtype mice	Intestinal stromal cells	Drop-seq	4,359	GSE116514	(Kim et al., 2020)
*Mus musculus*	Intestine	Endoderm	Definitive endoderm cells; trophectoderm cells; parietal endoderm cells; visceral endoderm cells; yolk sac endoderm cells	10X Chromium	112,217	GSE123046	(Nowotschin et al., 2019)
*Mus musculus*	Intestine	Embryo of wildtype mice	Epithelial and mesenchymal cells	STRT-seq	217	GSE87038	(Dong et al., 2018)
*Mus musculus*	Intestine	Wildtype and irradiated mice	Epithelial cells	10X Chromium	2,329	GSE145866	(Sheng et al., 2020)
*Mus musculus*	Small intestinal organoids	Wildtype and Rfx6 conditional deletion organoids	Epithelial cells	BD™ Precise WTA Single Cell Kit	290	GSE133038	(Piccand et al., 2019)
*Mus musculus*	Small intestinal organoids	Healthy and cancerous organoids	Epithelial cells	Multivariate-barcoded MC	> 1 million	https://community.cytobank.org/cytobank/login#project-id=1271	(Qin et al., 2020)
*Mus musculus*	Organoids	Lgr5^+^ cell-derived organoids	EECs	CEL-seq2	384	GSE114988	(Beumer et al., 2018)
*Mus musculus*	Crypts and organoids	Wildtype mice and intestinal organoids	Epithelial cells	CEL-seq	238	GSE62270	(Grun et al., 2015)
*Mus musculus*	Neurog3Chrono reporter mice and organoids	Neurog3 labeled mice	EECs	SORT-seq	6,906	GSE113561	(Gehart et al., 2019)
*Mus musculus*	Organoids	Lgr5^+^ and Lgr5^−^ single cells	Epithelial cells	10X Chromium	23,421	GSE115956	(Serra et al., 2019)
*Mus musculus*	Organoids upon RNAi-mediated APC and organoids transplanted into mice	APC mutated cells	Epithelial cells	C1	200	https://figshare.com/articles/dataset/Single_Cell/14518056	(Ono et al., 2021)
*Mus musculus*	Organoid	Organoids treated with forskolin, CFTRinh-172, or DMSO	Epithelial cells	inDrops	18,303	GSE164638	(Tallapragada et al., 2021)
*Mus musculus*	Small intestinal organoids	5-day differentiated BMP-off and BMP-on organoids	Epithelial cells	SORT-seq	Not described	GSE194004	(Beumer et al., 2022)
*Mus musculus*	Transgene-inducible intestinal organoids	Induced organoids for KRAS, BRAF and CTNNB1	Epithelial cells	BD™ Precise WTA Single Cell Kit	160,000	GSE115242	(Brandt et al., 2019)
*Mus musculus*	Small intestine and organoids	Wildtype mice and organoids	Epithelial cells	10X Chromium	10,180	GSE100274	(Mead et al., 2018)
*Mus musculus*	Intestinal adenoma of Apc^Min/+^mice at various time points	Colorectal cancer	Epithelial and mesenchymal cells	10X Chromium	79,801	GSE136256	(Zowada et al., 2021)
*Homo sapiens*	Ileum	CD tissues	Lamina propria cells	10X Chromium	82,417	GSE134809	(Martin et al., 2019)
*Homo sapiens*	Ileum	Health and CD tissues	Immune cells	10X Chromium	16,731	GSE157477	(Jaeger et al., 2021)
*Homo sapiens*	Esophagus, stomach, duodenum	Healthy tissues	Epithelium; immune cells	SORT-seq	Not described	GSE157694/EGAS00001004695	(Busslinger et al., 2021)
*Homo sapiens*	Small intestine	Donor- and recipient-derived cells after transplantation	T cells	10X Chromium	974 for scRNA-seq,196 for smart-seq	GSE162687	(FitzPatrick et al., 2021)
*Homo sapiens*	Small intestine	Healthy tissues	Epithelial cells	10X Chromium	12,590	GSE185224	(Burclaff et al., 2022)
*Homo sapiens*	Ileum and ileum-derived organoids	Healthy and viral infected tissues	Epithelial cells	10X Chromium	25,482	GSE171620	(Triana et al., 2021)
*Homo sapiens*	Colon	Healthy and IBD tissues	Epithelial and mesenchymal cells	Smart-seq2	11,175	GSE116222	(Parikh et al., 2019)
*Homo sapiens*	Colon	Healthy and UC tissues	Epithelial cells & mesenchymal cells & immune cells	10X Chromium	366,650	SCP259	(Smillie et al., 2019)
*Homo sapiens*	Colon	Colorectal cancer	Cancer cells, stromal cells, and immune cells	10X Chromium	27,927	GSE188711	(Guo et al., 2022)
*Homo sapiens*	Colon	Colorectal cancer	Immune cells	10X Chromium	178,630	OEP001756	(Wu et al., 2022)
*Homo sapiens*	Colon	Tumors and adjacent tissues	T cells	10X Chromium	37,931	EMTAB-9455	(Masuda et al., 2022)
*Homo sapiens*	Colon	Healthy and UC tissues	Immune cells, stromal cells	10X Chromium	29,046	GSE182270	(Uzzan et al., 2022)
*Homo sapiens*	Colon	Healthy tissues	Macrophages	10X Chromium	63,970	EGAD00001007765/EGAS00001005377	(Domanska et al., 2022)
*Homo sapiens*	Colon and rectum	Familial adenomatous polyposis	Environmental cells (endothelial cells, fibroblasts, macrophages, mast cells, T cells and B cells) and epithelial cells	STRT-Seq (modified)	8,757	Not described	(Li et al., 2020)
*Homo sapiens*	Large intestine	Children with undifferentiated colitis, Crohn’s disease, and ulcerative colitis	Epithelium, mesenchyme and immune cells	10X Chromium	73,165	GSE121380	(Huang et al., 2019)
*Homo sapiens*	Large intestine	Colorectal cancer	Immune and non-immune cells	10X Chromium and Smart-seq2	43,817 (Hi-seq 4000); 10,468 (Smart-seq2)	GSE146771	(Zhang et al., 2020a)
*Homo sapiens*	Large intestine	Healthy; melanoma with Checkpoint inhibitor-induced colitis; melanoma without Checkpoint inhibitor-induced colitis	Immune cells	10X Chromium	51,652	GSE144469	(Luoma et al., 2020)
*Homo sapiens*	Large intestine	Healthy, CD and UC tissues	CD45^+^ immune cell	10X Chromium	63,314	PRJCA003980	(Huang et al., 2021)
*Homo sapiens*	Large intestine	Healthy and UC tissues	Immune cells	10X Chromium	20,678(UC) 16,678 (Health)	GSE162335	(Devlin et al., 2021)
*Homo sapiens*	Ileum, colon, rectum	Precancerous tissues	Epithelial cells	10X Chromium	14,537	GSE125970	(Wang et al., 2020b)
*Homo sapiens*	Small intestine, large intestine	Fetal digestive tissues	Epithelial and mesenchymal cells	STRT-seq	5,227	GSE103239	(Gao et al., 2018)
*Homo sapiens*	Embryonic gastrointestinal tract	Healthy tissues	Epithelial cells	Not described	5,290	Supplementary Table 1 and 2	(Tan et al., 2019)
*Homo sapiens*	Human embryonic intestinal tract	Healthy tissues	Epithelial, mesenchymal and immune cells	10X Chromium	24,783	E-MTAB-9489	(Holloway et al., 2021)
*Homo sapiens*	Human embryonic and adult intestinal tract	Healthy tissues	Epithelial, mesenchymal and immune cells	10X Chromium	428,000	E-MTAB-9543, E-MTAB-9536, E-MTAB-9532, E-MTAB-9533 and E-MTAB-10386	(Elmentaite et al., 2021)
*Homo sapiens*	Small intestinal organoids	Crohn’s disease patient-derived tissues	Epithelial cells	C1	1,037	Not described	(Suzuki et al., 2018)
*Homo sapiens*	Human pluripotent stem cell-derived intestinal organoids	Organoids	Epithelium, mesenchyme, endothelium, and neurons	10X Chromium	13,289	E-MTAB-9228	(Holloway et al., 2020)
*Homo sapiens*	Intestine and organoids	Intestinal tissues and organoids	EECs	10X Chromium	15,283	GSE146799	(Beumer et al., 2020)
*Homo sapiens*	Cell lines	Colorectal cancer	SW480 cells	10X Chromium & SORT-seq	192	Not described	(Yi et al., 2020)
*Homo sapiens*	Cell lines	Control and 5FU-treated colon cancer	5FU-treated RKO, HCT116, SW480	Drop-seq	10,421	GSE149224	(Park et al., 2020)
*Homo sapiens*	Large intestine and cell lines	Healthy tissues and colorectal cancer	Epithelial cells	C1	2,221	GSE81861	(Wu et al., 2017)
*Homo sapiens*	Primary and metastasis tumor	Colorectal cancer	Spheroids, Tumors, PDXs, PDOs	10X Chromium and iCELL8	26,170	EGAS00001004064	(Zowada et al., 2021)
*Homo sapiens*	Tumors and adjacent normal tissues	Colorectal cancer	T cells	Smart-seq2	8,530	EGAS00001002791 / GSE108989	(Zhang et al., 2018)
*Homo sapiens*	Embryonic, fetal, childhood/adolescence ileum	Healthy and pediatric Crohn’s disease tissues	Epithelial, mesenchymal and immune cells	10X Chromium	74,106	E-MTAB-8901	(Elmentaite et al., 2020)
*Homo sapiens*	Human embryonic intestinal tract	Healthy tissues	Epithelial, mesenchymal and immune cells	10X Chromium	76,592	GSE158328/GSE158702	(Fawkner-Corbett et al., 2021)
*Mus musculus; Homo sapiens*	Intestine and organoids	Colonic mesenchyme; fibroblast-crypt cocultures; Ptger4-knockout crypt epithelial cells	Epithelial cells and mesenchymal cells	Drop-seq	5,371	GSE142431	(Roulis et al., 2020)
*Mus musculus; Homo sapiens*	Colon	Healthy, UC and DSS treated tissues	Epithelial and mesenchymal cells	10X Chromium	11,549	GSE114374	(Kinchen et al., 2018)
*Mus musculus; Homo sapiens*	Human embryonic intestinal tract and mouse colon and colitis	Healthy and DSS treated tissues	Epithelial, mesenchymal and immune cells	10X Chromium	22,579	GSE151257/GSE154007	(Fazilaty et al., 2021)
*Mus musculus; Homo sapiens*	Intestinal organoids	Organoids	Epithelial cells	DisCo	945	GSE148093	(Bues et al., 2022)
*Rhesus macaques*	Large intestine	Healthy and during acute graft-versus-host disease tissues	T cells	10X Chromium	21,490	GSE142483	(Tkachev et al., 2021)
*Zebrafish*	Whole intestine	Healthy and DSS treated tissues	Epithelial, mesenchymal and immune cells	10X Chromium	30,069	https://github.com/Cho-lab-Sinai/Scripts_Nayar_et_al	(Nayar et al., 2021)
*Swine*	Ileum	6 time points in the swine neonatal period	Epithelial cells	10X Chromium	40,186	GSE162287	(Meng et al., 2021)
*Drosophila melanogaster*	Midgut	Healthy tissues	EECs	10X Chromium	4,661	GSE132274	(Guo et al., 2019)

Nowadays, we can perform single-cell sequencing analysis with millions of cells (Qin et al., 2020), but the sequencing depth in each cell is limited in the massive scRNA-seq. On another side, some sequencing technologies, such as Smart-seq2 (Picelli et al., 2013), shows a great sequencing depth, but can only apply to small cell number due to the cost. If the depth sequencing can be combined with massive single-cell numbers cost-effectively, more information will be gained (Lai et al., 2020). Furthermore, other single-cell technologies are under development, such as single-cell protein mass spectrum, single-cell sequencing of whole epigenomics, and combinatorial DNA/RNA/epigenetic sequencing in one cell (Bian et al., 2018; Hutchins, 2021), the low-input but high-resolution analysis scRNA-seq methods (Bues et al., 2022). If the breakthroughs of these technologies on the scale, sensitivity, and cost-efficiency can be made, biomedical research will enter the single-cell era.

## Data Availability

Not applicable.

## Abbreviations

5-HT: 5-Hydroxytryptamine
BMP: Bone morphogenetic protein
CCK: Cholecystokinin
CD: Crohn’s disease
ChgA: Chromogranin-A
CPI: Checkpoint inhibitor-induced colitis
CRC: Colorectal cancer
DISCO: Digital microfluidic isolation of single cells for-omics
DSS: Dextran sulphate sodium
EECs: Enteroendocrine cells
EGF: Epidermal growth factor
EMT: Epithelial-to-mesenchymal transition
FACS: Fluorescence activated cell sorter
Foxl1: Forkhead box L1
GAST: Gastrin
GCG: Glucagon
GHRL: Ghrelin
GIP: Glucose-dependent insulinotropic polypeptide
HIST: Histamine
IBD: Inflammatory bowel disease
icGCs: Intercrypt goblet cells
ISCs: Intestinal stem cells
JAK: Janus kinase
LCM-RNA-seq: RNA-sequencing of laser capture micro-dissected tissue
MARS-seq: Massively parallel single-cell RNA-sequencing
MLN: Motilin
NTS: Neurotensin
PSMA6: Proteasome 20S subunit alpha 6
PYY: Peptide YY
scDNA-seq: Single-cell DNA sequencing
scRNA-seq: Single-cell RNA sequencing
SCT: Secretin
SORT-seq: FACS sorting followed by single-cell RNA-sequencing
SST: Somatostatin
STRT-seq: Single-cell tagged reverse transcription sequencing
TA cells: Transient-amplifying cells
TAC1: Tachykinin 1
TCR: Toll-like receptor
Th2 cells: Type 2 helper T cells
TNF: Tumor necrosis factor
UC: Ulcerative colitis
